# Associations between ambient temperature and adult asthma hospitalizations in Beijing, China: a time-stratified case-crossover study

**DOI:** 10.1186/s12931-022-01960-8

**Published:** 2022-02-22

**Authors:** Yuxiong Chen, Dehui Kong, Jia Fu, Yongqiao Zhang, Yakun Zhao, Yanbo Liu, Zhen’ge Chang, Yijie Liu, Xiaole Liu, Kaifeng Xu, Chengyu Jiang, Zhongjie Fan

**Affiliations:** 1 Department of Medicine, Peking Union Medical College Hospital, Peking Union Medical College & Chinese Academy of Medical Sciences, No.1 Shuaifuyuan Wangfujing Dongcheng District,, Beijing, 100730 China; 2 Department of Biochemistry, State Key Laboratory of Medical Molecular Biology, Institute of Basic Medical Sciences, Chinese Academy of Medical Sciences, Peking Union Medical College, Beijing, 100005 China

**Keywords:** Ambient temperature, Asthma, Hospitalizations, Heat effect, Cold effect

## Abstract

**Background:**

Studies on the associations between ambient temperature and asthma hospitalizations are limited, and the results are controversial. We aimed to assess the short-term effects of ambient temperature on the risk of asthma hospitalizations and quantify the hospitalization burdens of asthma attributable to non-optimal temperature in adults in Beijing, China.

**Methods:**

We collected daily asthma hospitalizations, meteorological factors and air quality data in Beijing from 2012 to 2015. We applied a time-stratified case-crossover design and fitted a distributed lag non-linear model with a conditional quasi-Poisson regression to explore the association between ambient temperature and adult asthma hospitalizations. The effect modifications of these associations by gender and age were assessed by stratified analyses. We also computed the attributable fractions and numbers with 95% empirical confidence intervals (eCI) of asthma hospitalizations due to extreme and moderate temperatures.

**Results:**

From 2012 to 2015, we identified a total of 18,500 hospitalizations for asthma among adult residents in Beijing, China. Compared with the optimal temperature (22 °C), the cumulative relative risk (CRR) over lag 0–30 days was 2.32 with a 95% confidence interval (CI) of 1.57–3.42 for extreme cold corresponding to the 2.5th percentile (− 6.5 °C) of temperature distribution and 2.04 (95% CI 1.52–2.74) for extreme heat corresponding to the 97.5th percentile (29 °C) of temperature distribution. 29.1% (95% eCI 17.5–38.0%) of adult asthma hospitalizations was attributable to non-optimum temperatures. Moderate cold temperatures yielded most of the burdens, with an attributable fraction of 20.3% (95% eCI 9.1–28.7%). The temperature-related risks of asthma hospitalizations were more prominent in females and younger people (19–64 years old).

**Conclusions:**

There was a U-shaped association between ambient temperature and the risk of adult asthma hospitalizations in Beijing, China. Females and younger patients were more vulnerable to the effects of non-optimum temperatures. Most of the burden was attributable to moderate cold. Our findings may uncover the potential impact of climate changes on asthma exacerbations.

**Supplementary Information:**

The online version contains supplementary material available at 10.1186/s12931-022-01960-8.

## Background

Asthma is among the most prevalent chronic airway diseases with significant public health consequences. According to the Global Burden of Disease, asthma affected about 358 million people in 2015, leading to an enormous disease burden worldwide [1]. In China, approximately 4.2% of the adult population is suffering from asthma, representing 45.7 million Chinese adults [2]. Asthma is characterized by reversible airflow obstruction with symptoms of coughing, wheezing, breathlessness and chest stuffiness [3]. As there is no definitive cure, the disease tends to recur and seriously affect patients’ quality of life. Considering the high prevalence of asthma and the difficulty in control and intervention, a comprehensive understanding of the risk factors of asthma exacerbations is imperative. Many environmental risk factors, such as air pollution, pollen, tobacco smoke and meteorological factors have been reported to contribute to asthma exacerbations, leading to hospitalization [4–6].

Among numerous meteorological factors, temperature has been the most studied [7]. Although some epidemiological studies have reported that both cold and heat exposures are associated with increased risks of asthma [8–10], the association between ambient temperature and asthma hospitalizations is still inconclusive. The majority of studies have only found significant cold effects on asthma hospital visits or admissions [11–16], while limited studies have provided evidence for increased risks of asthma caused by heat exposures [17–19]. With global climate changes, extreme weather events, especially heatwaves, are expected to increase in frequency and intensity [20]. The growing public concern about a warming climate urges health practitioners to further clarify the relationships between potential disease risks and heat exposures. Moreover, most previous studies only quantified the association between asthma and ambient temperature by ratio measures, such as relative risk or odds ratio. Yet, few studies have estimated the attributable burden as well [21, 22]. Compared with ratio measures, the attributable burden may provide more specific information on the actual influence of the exposures and benefits of prevention and intervention by calculating attributable risk measures, involving attributable fraction (AF) and attributable number (AN) [23].

The study aimed to explore the exposure–response relationship between ambient temperature and daily hospitalizations for asthma among adults in Beijing, China, and how the relationship varied within different sex and age groups. We also calculated the total hospitalization burden of asthma attributed to non-optimum temperature and the relative contributions separated into different temperature ranges, including extreme heat, moderate heat, moderate cold and extreme cold. Our findings may assist in developing suitable public health interventions and risk assessment methods for reducing asthma exacerbations resulting from abnormal ambient temperatures.

## Methods

### Data source

Our study area was Beijing, which is located in north China (39° 56′ N, 116° 20′ E). As the capital city of China, it covers an area of 16,410.54 km^2^, with more than 21 million residents in 2015. Beijing’s climate belongs to a temperate semi-humid continental monsoon climate characterized by hot, rainy summer and cold, dry winter. In 2015, a cross-sectional study conducted in Beijing among 26,166 Chinese adults aged 20 years or older showed that the prevalence of doctor-diagnosed asthma was 2.8% in males and 2.1% in females [24].

We obtained daily records of hospitalizations for asthma (emergency department or outpatient visits were not included) from January 1, 2012 to December 31, 2015 from the Beijing Public Health Information Center (http://www.phic.org.cn/). The center collected inpatient data from all the tertiary and secondary hospitals in Beijing by the electronic disease reporting system. Each record contained the hospital’s name, date of admission and discharge, patient’s gender, date of birth, residential address, and discharge diagnoses. The hospitalization data included in our study were identified by asthma as a primary discharge diagnosis according to the 10th version of the International Classification of Diseases (ICD-10, code J45-46). Further, individuals residing outside of Beijing or aged ≤ 18 years old were excluded from this study. The study was approved and exempt from the full ethical review by the Institutional Review Board of Peking Union Medical College Hospital because all the records included were anonymous.

We acquired daily meteorological data during the same period from the China Meteorological Data Sharing Service System (http://data.cma.cn/), including daily minimum, maximum, and mean temperature (°C), mean relative humidity (%), and mean wind speed (m/s). To adjust for the potential effects of air pollutants, daily air quality index (AQI) data were extracted from the China National Environmental Monitoring Centre (http://www.cnemc.cn/). The daily AQI value is a comprehensive indicator of air quality, determined by the maximum value of individual air quality indexes of six regularly monitored air pollutants (fine particulate matter, inhalable particulate matter, sulfur dioxide, nitrogen dioxide, ozone, and carbon monoxide).

Since influenza has been associated with asthma exacerbations [25], we also collected information on influenza epidemics defined as the proportion of isolates positive for influenza surpassed 30% of the maximum seasonal level (Influenza surveillance season was defined from the 27th week of the previous year to the 26th week of the following year) [26] from the Chinese National Influenza Center (http://www.chinaivdc.cn/cnic/).

### Study design and data analysis

We used a time-stratified case-crossover design to estimate the association between ambient temperature and daily hospitalizations for asthma in adults. In contrast with the traditional case–control study, the case-crossover design has superiority in controlling the time-invariant confounders of individual characteristics because each person serves as his or her own control. For each admission, the case day was specified as the admission date. To minimize the bias of long-term trend, seasonality, and “weekday effects”, we matched 3 or 4 control days by the same day of the week earlier or later within the same month and year to the case days. A conditional quasi-Poisson regression model was applied to allow for over-dispersion in daily counts of asthma hospitalizations [27].

Previous studies have shown that the relationships between ambient temperature and asthma hospitalizations were non-linear with a lagged effect [8, 11]. Thus, a distributed lag non-linear model (DLNM) was adopted to assess the complicated non-linear and delayed temperature-health dependencies simultaneously [28] after adjusting for relative humidity, wind speed, AQI and influenza epidemics. We modeled the temperature exposure–response with a natural cubic spline with 4 degrees of freedom (df) and the lag-response with a natural cubic spline with 3 df in the log scale. We set a maximum lag of 30 days to explore the long delay of the effects because the effects of cold temperatures could last about 2–3 weeks with no substantial effects after more than 1 month [29], which was consistent with previous studies [8, 11, 30]. Although the effects of hot temperatures were more acute, the potential morbidity displacement by harvesting effects of high temperatures should be noticed [30]. Moreover, the possible delayed admission time should be considered because our study focused on hospitalizations for asthma rather than outpatient hospital visits or emergency department visits. The final model was as follows:

$$Log\left[ {E\left( {Y_{t} } \right)} \right] = \beta Temp_{t,l} + ns\left( {RH_{t} ,df = 4} \right) + ns\left( {WS_{t} ,df = 3} \right) + ns\left( {AQI_{t} ,df = 4} \right) + factor\left( {stratum} \right) + \gamma Influenza_{t} + \alpha$$

where t is the day of observation; E(Y_t_) denotes the expected daily counts of asthma hospitalizations on day t. Temp_t,l_ is the cross-basis matrix to the temperature generated by DLNM. l represents the lag days. ns() is the natural cubic spline-smoothing function for non-linear variables, including RH, relative humidity; WS, wind speed, and AQI, air quality index. df means degrees of freedom. The optimal df selection for variable terms was based on minimizing the quasi-Poisson Akaike information criteria of the model. The degrees of freedom for relative humidity, wind speed, and AQI were set at 4, 3, 4, respectively. Stratum refers to the matched groups of case days and control days. Influenza_t_ is a binary variable. If the day t is in the period of an influenza epidemic, the value of 1 otherwise 0. β and γ are the vectors of coefficients for corresponding variables. α is the model intercept.

We performed subgroup analyses stratified by gender and age (19–64 years old and ≥ 65 years old) to examine their potential modification effects. The minimum admission temperature (MAT) was determined by finding the temperature value corresponding to the lowest risk of total adult asthma hospitalizations from the cumulative exposure–response curve. We also took this value as the optimum temperature for all the subgroups. To quantitatively evaluate the effects of extreme heat and cold exposures, we calculated the relative risk (RR) and 95% confidence interval (CI) at the extreme values (the 97.5th percentile and the 2.5th percentile of daily mean temperature) relative to the MAT [31, 32]. The statistical methods of the study have been widely used in estimating the health impact of ambient temperature [33–35].

To quantify the AF and AN due to non-optimum temperatures under the framework of DLNM, we employed a backward perspective strategy that estimated the risk at time t as attributable to a series of past exposure events [23]. The formulas of backward AF_x,t_, and AN_x,t_ at time t were as follows:

$$\mathrm{b}-{AF}_{x,t}=1-\mathrm{exp}\left(\sum_{l={l}_{0}}^{L}{\upbeta x}_{t-l}\text{,}l\right)$$

$$\mathrm{b}-{AN}_{x,t}=\mathrm{b}-{AF}_{x,t}\cdot {n}_{t}$$

where n_t_ denotes the count of cases at time t. AN_x,t_ and AF_x,t_ implies the number of cases and the corresponding fraction at time t attributable to past exposures to x in the period t − l_0_,…, t − L. We further separated the temperature distribution into four components including the extreme cold and heat, and the moderate cold and heat [21, 31]. The extreme heat and cold were defined as temperatures higher than the 97.5th percentile and lower than the 2.5th percentile, respectively. The moderate heat and cold were defined as the ranges between the MAT and the 97.5th and 2.5th percentiles, respectively. Through Monte Carlo simulations, the estimation of 95% empirical confidence intervals (eCI) for AF and AN was determined as the related 2.5th and 97.5th percentiles of distributions by simulating 5000 random samples.

All statistical analyses were conducted using R software (version 3.6.1) with the packages of “gnm”, “dlnm” and “attrdl”. Statistical significance was regarded as a two-sided *p* < 0.05.

### Sensitivity analysis

To test the robustness of our results, we conducted a series of sensitivity analyses: (1) changing df for relative humidity (3–5 df), wind speed (3–5 df) and AQI (3–5 df); (2) altering the df (3–5) of the natural cubic spline in the log scale for the lag-response space; (3) changing the maximum lag days to 14 and 21 in the lag parameter in DLNM; (4) applying different temperature metrics, including daily minimum temperature, maximum temperature and mean apparent temperature. The apparent temperature (AT) is an aggregative indicator to reflect the human thermal perception of ambient temperature. The indicator combines outdoor temperature with relative humidity and wind velocity. We calculated the daily mean AT using the following equations:

1 $${\text{AT}} = {\text{T}} + 0.{33} \times {\text{e}} - 0.{7}0 \times {\text{WS}} - {4}.00$$

2 $${\text{e}} = {\text{RH}}/{1}00 \times {6}.{1}0{5} \times {\text{exp}}\left[ {{17}.{27} \times {\text{T}}/\left( {{237}.{7} + {\text{T}}} \right)} \right]$$

where T denotes daily mean temperature (°C), WS refers to wind speed (m/s), e means water vapor pressure (hPa) which was calculated with Eq. (2); RH is relative humidity (%).

## Results

### Data description

**Fig. 1:**
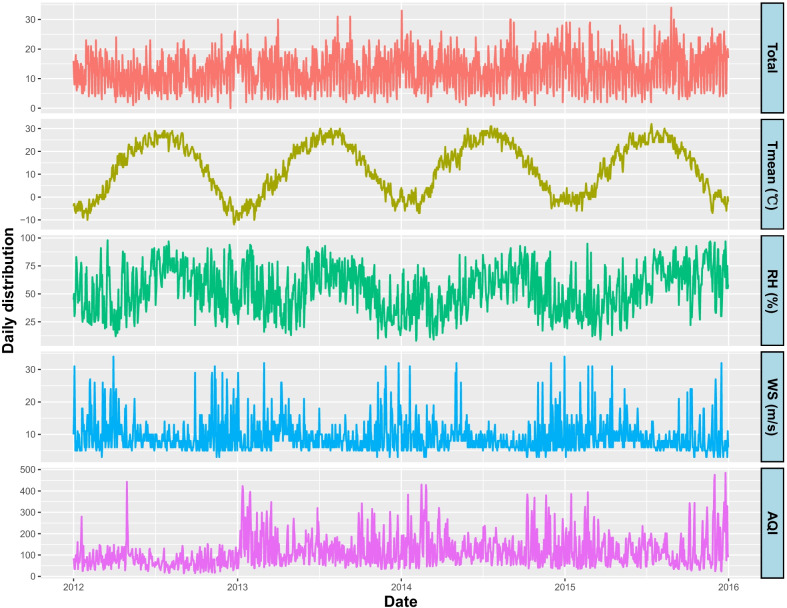
Time-series plots for daily adult asthma hospitalizations, meteorological variables and air quality in Beijing, 2012–2015. (Tmean: mean temperature (°C), RH: relative humidity (%), WS: wind speed (m/s), AQI: air quality index)

Table 1 provides the descriptive statistics for daily adult asthma hospitalizations, meteorological variables and air quality from January 1, 2012 to December 31, 2015. During the study period, a total of 18,500 hospitalizations for asthma among adult residents were recorded in Beijing, a mean of 12.7 per day (range, 0–34). Of all these cases, there were more asthma hospitalizations for females (59.3%) than for males (40.7%). People aged 19–64 had more asthma hospitalizations than people aged 65 or above (59.9% vs. 40.1%). The median (minimum, maximum) values of daily mean temperature, minimum temperature, maximum temperature and mean apparent temperature were 14.0 °C (− 12.0 °C, 32.0 °C), 8.0 °C (− 18.0 °C, 27.0 °C), 21.0 °C (− 7.0 °C, 42.0 °C) and 6.2 °C (− 34.1 °C, 32.7 °C), respectively. The medians of relative humidity, wind speed and AQI were 54.0% (range, 8.0–98.0%), 8.0 m/s (range, 3.0–34.0 m/s) and 94.0 (range, 14.0–485.0), respectively. Figure 1 shows the time-series plots of daily meteorological variables, AQI and hospitalizations for asthma among adults in Beijing, 2012–2015.

**Table 1:** Descriptive statistics of daily adult asthma hospitalizations, meteorological variables and air quality in Beijing, China from 2012 to 2015

	n (%)	Mean ± SD	Minimum	Percentiles			Maximum
				25th	50th	75th	
Asthma hospitalizations							
Total	18,500 (100)	12.7 ± 6.1	0.0	8.0	13.0	17.0	34.0
Male	7522 (40.7)	5.1 ± 3.0	0.0	3.0	5.0	7.0	18.0
Female	10,978 (59.3)	7.5 ± 4.1	0.0	4.0	7.0	10.0	26.0
19–64 years old	11,079 (59.9)	7.6 ± 4.0	0.0	5.0	7.0	10.0	24.0
≥ 65 years old	7421 (40.1)	5.1 ± 3.2	0.0	3.0	5.0	7.0	18.0
Environmental variables							
Mean temperature (°C)	/	12.6 ± 11.3	− 12.0	2.0	14.0	23.0	32.0
Minimum temperature (°C)	/	6.9 ± 11.4	− 18.0	− 3.0	8.0	18.0	27.0
Maximum temperature (°C)	/	18.6 ± 11.6	− 7.0	7.0	21.0	29.0	42.0
Mean apparent temperature (°C)	/	5.5 ± 14.9	− 34.1	− 7.8	6.2	19.1	32.7
Relative humidity (%)	/	53.9 ± 20.1	8.0	38.0	54.0	70.0	98.0
Wind speed (m/s)	/	9.4 ± 4.9	3.0	6.0	8.0	11.0	34.0
Air quality index	/	115.5 ± 73.1	14.0	65.0	94.0	147.0	485.0

*SD* standard deviation

### Associations between ambient temperature and adult asthma hospitalizations

**Fig. 2:**
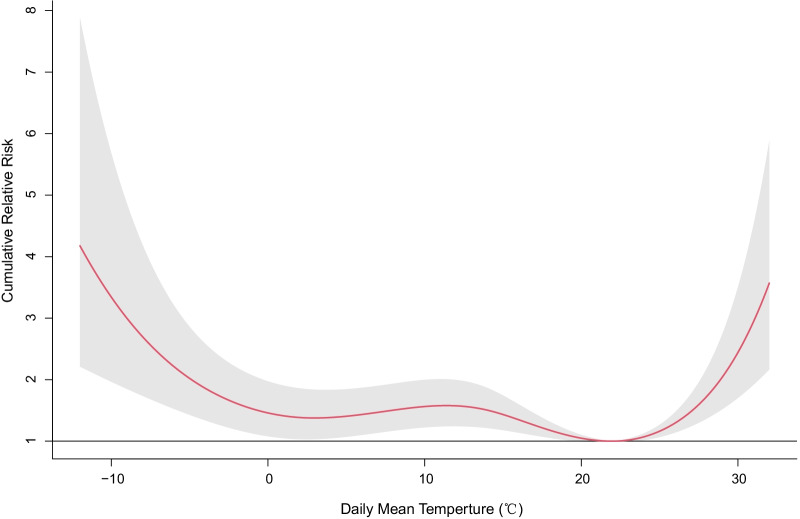
Lag-cumulative exposure–response associations between daily mean temperature and total adult asthma hospitalizations over lag 0–30 days in Beijing, 2012–2015

Figure 2 shows the cumulative exposure–response curve exhibited a U-shaped relationship between daily mean temperature and total adult asthma hospitalizations across a 30-day lag period. For the whole study population, the MAT was 22 °C. Cumulative relative risk (CRR) of hospitalizations for asthma increased significantly below and above the MAT.

**Fig. 3:**
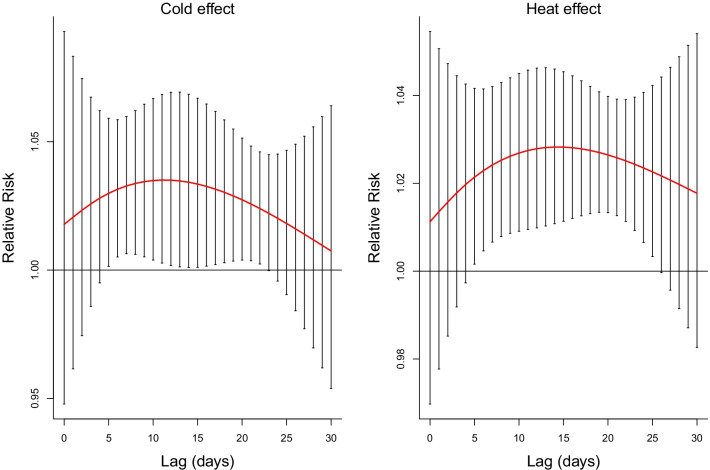
Lag-response relationships between extreme cold exposure [2.5th percentile (− 6.5 °C) relative to MAT (22 °C)] and heat exposure [97.5th percentile (29 °C) relative to MAT] and total adult asthma hospitalizations in Beijing, 2012–2015. (*MAT* minimum admission temperature)

Table 2 shows the cumulative effects of extreme temperatures on total adult asthma hospitalizations over different lag periods (0, 0–3, 0–7, 0–14, 0–21, and 0–30 days, i.e. the associations between single-day temperature exposures and the cumulative risks of asthma hospitalizations from the same day (lag 0) to the next few days). We found that both extreme cold (2.5th percentile vs. MAT) and heat (97.5th percentile vs. MAT) exposures were significantly associated with higher risks of asthma hospitalizations through lag 14 to 30 days, with the maximum CRR over lag 0–30 days (Extreme cold: CRR = 2.32, 95% CI 1.57, 3.42; Extreme heat: CRR = 2.04, 95% CI 1.52, 2.74). The CRRs associated with extreme cold were higher than those associated with extreme heat. Figure 3 shows the lag-response relationships between extreme temperatures and total adult asthma hospitalizations over lag 0–30 days. We observed that both the lagged effects of extreme cold and heat lasted for about 3 or 4 weeks. The extreme cold effects became significant on lag day 5 and lasted until lag day 22, whereas the extreme heat effects became significant on lag day 5 and persisted until lag day 25.

**Table 2:** Lag-cumulative relative risks for total adult asthma hospitalizations associated with extreme cold exposure [2.5th percentile (− 6.5 °C) relative to MAT (22 °C)] and extreme heat exposure [97.5th percentile (29 °C) relative to MAT]

Lag days	Cold effects	Heat effects
	CRR (95% CI)	CRR (95% CI)
Lag 0	1.02 (0.95, 1.10)	1.01 (0.97, 1.05)
Lag 0–3	1.10 (0.88, 1.37)	1.06 (0.93, 1.21)
Lag 0–7	1.25 (0.92, 1.70)	1.16 (0.95, 1.41)
Lag 0–14	1.61 (1.12, 2.31)*	1.39 (1.09, 1.79)*
Lag 0–21	1.99 (1.32, 3.02)*	1.68 (1.27, 2.23)*
Lag 0–30	2.32 (1.57, 3.42)*	2.04 (1.52, 2.74)*

*MAT* minimum admission temperature, *CRR* cumulative relative risk, *CI* confidence interval

*p < 0.05

**Fig. 4:**
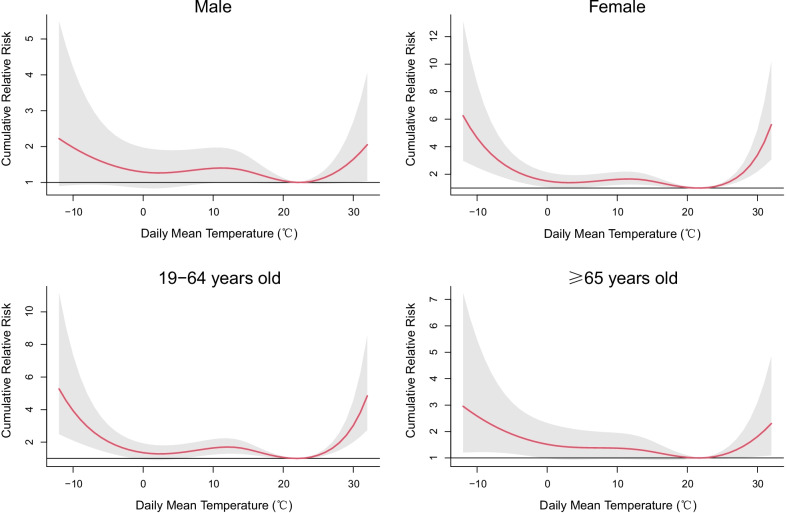
Lag-cumulative exposure–response associations between daily mean temperature and adult asthma hospitalizations over lag 0–30 days stratified by gender and age in Beijing, 2012–2015

Figure 4 shows the exposure–response plots for the subgroups over lag 0–30 days. The variation trends of CRR in all the subgroups followed a similar pattern as the total study population. Table 3 shows the lag-cumulative effects of extreme cold and heat exposures by subgroup analyses stratified by gender and age at different lag days. Among all the subgroups, the risks of extreme cold on adult asthma hospitalizations were higher than those of extreme heat. Significant associations between both the extreme cold and heat exposures and adult asthma hospitalizations in females appeared at lag times of 14–30 days. However, no significant association was detected in males. Female asthma patients seem to be more vulnerable to extreme temperatures than male patients. Regarding different age groups, the cumulative effects of extreme temperatures (both heat and cold) in the younger patients (19–64 years old) were significant at lag times of 14–30 days, while the cumulative effects were significant only at a lag of 30 days in the elderly (≥ 65 years old). The CRRs of extreme cold and heat exposures over lag 0–30 days were higher in the younger group (Extreme cold: CRR = 2.46, 95% CI 1.56, 3.89; Extreme heat: CRR = 2.39, 95% CI 1.71, 3.34) than the elderly (Extreme cold: CRR = 2.11, 95% CI 1.22, 3.64; Extreme heat: CRR = 1.56, 95% CI 1.00, 2.43). The results suggested that the younger patients were more likely to be exposed to higher risk in extreme temperatures.

**Table 3:** Lag-cumulative relative risks of adult asthma hospitalizations associated with extreme cold exposure [2.5th percentile (− 6.5 °C) relative to MAT (22 °C)] and heat exposure [97.5th percentile (29 °C) relative to MAT] stratified by gender and age

Lag days	Cold effects				Heat effects			
	CRR (95% CI)				CRR (95% CI)			
	Male	Female	19–64 years old	≥ 65 years old	Male	Female	19–64 years old	≥ 65 years old
Lag 0	1.01 (0.91, 1.12)	1.03 (0.94, 1.12)	0.99 (0.91, 1.08)	1.06 (0.96, 1.17)	1.01 (0.96, 1.07)	1.01 (0.96, 1.06)	1.03 (0.98, 1.08)	0.98 (0.92, 1.04)
Lag 0–3	1.05 (0.77, 1.44)	1.14 (0.88, 1.47)	1.04 (0.80, 1.35)	1.18 (0.87, 1.61)	1.05 (0.87, 1.26)	1.07 (0.91, 1.26)	1.12 (0.96, 1.31)	0.96 (0.79, 1.17)
Lag 0–7	1.12 (0.73, 1.73)	1.34 (0.94, 1.91)	1.26 (0.88, 1.81)	1.22 (0.80, 1.87)	1.09 (0.84, 1.43)	1.21 (0.96, 1.53)	1.26 (1.01, 1.59)*	1.00 (0.75, 1.33)
Lag 0–14	1.29 (0.78, 2.15)	1.85 (1.21, 2.83)*	2.02 (1.32, 3.09)*	1.16 (0.70, 1.93)	1.17 (0.83, 1.65)	1.59 (1.18, 2.15)*	1.55 (1.16, 2.06)*	1.17 (0.81, 1.69)
Lag 0–21	1.47 (0.82, 2.64)	2.43 (1.49, 3.95)*	2.80 (1.72, 4.58)*	1.23 (0.68, 2.22)	1.27 (0.87, 1.86)	2.08 (1.49, 2.91)*	1.88 (1.37, 2.59)*	1.39 (0.92, 2.10)
Lag 0–30	1.64 (0.94, 2.84)	2.90 (1.85, 4.54)*	2.46 (1.56, 3.89)*	2.11 (1.22, 3.64)*	1.47 (0.99, 2.21)	2.60 (1.82, 3.70)*	2.39 (1.71, 3.34)*	1.56 (1.00, 2.43)*

*MAT* minimum admission temperature, *CRR* cumulative relative risk, *CI* confidence interval

*p < 0.05

### Attributable burden of adult asthma hospitalizations due to ambient temperature

Table 4 shows the estimated AF and AN of adult asthma hospitalizations owing to both cold and heat exposures divided into contributions from moderate and extreme temperatures. The total fraction of hospitalization for asthma among adults caused by non-optimal temperatures over lag 0–30 days was 29.1% (95% eCI 17.5%, 38.0%), and most of them were related to the moderate cold (20.3%, 95% eCI 9.1%, 28.7%). Both extreme heat and cold exposures were responsible for a small portion, with the AF equal to 2.2% (95% eCI 1.3, 3.0) for extreme heat and 2.4% (95% eCI 1.4, 3.1) for extreme cold.

**Table 4:** Attributable fraction (%) and attributable number (95% eCI) of adult asthma hospitalizations due to different temperature ranges in the total population and subgroups stratified by gender and age

Group	Overall		Extreme heat		Moderate heat		Moderate cold		Extreme cold	
	AF (%)	AN	AF (%)	AN	AF (%)	AN	AF (%)	AN	AF (%)	AN
Total	29.1 (17.5, 38.0)	5379 (3297, 7038)	2.2 (1.3, 3.0)	404 (247, 547)	5.5 (3.0, 7.8)	1017 (550, 1424)	20.3 (9.1, 28.7)	3750 (1755, 5337)	2.4 (1.4, 3.1)	437 (255, 585)
Male	19.9 (1.0, 33.5)	1499 (43, 2478)	1.3 (0, 2.4)	95 (− 2, 181)	3.1 (− 1.0, 6.6)	233 (− 74, 496)	14.6 (− 2.8, 27.0)	1097 (− 236, 1997)	1.4 (− 0.3, 2.7)	109 (− 20, 202)
Female	34.5 (21.6, 44.2)	3791 (2362, 4843)	2.8 (1.8, 3.7)	307 (202, 406)	7.0 (4.3, 9.5)	768 (477, 1035)	23.8 (11.3, 32.9)	2607 (1237, 3623)	2.9 (1.9, 3.7)	317 (198, 410)
19–64 years old	30.9 (18.1, 40.7)	3423 (1964, 4469)	2.8 (1.8, 3.7)	308 (196, 408)	6.9 (4.0, 9.5)	763 (442, 1048)	20.2 (7.9, 29.4)	2233 (824, 3231)	2.4 (1.3, 3.3)	269 (146, 367)
≥ 65 years old	26.0 (7.7, 39.4)	1889 (502, 2872)	1.3 (0.1, 2.4)	94 (3, 174)	3.3 (− 0.5, 6.5)	239 (− 31, 473)	20.1 (2.2, 32.9)	1464 (186, 2377)	2.1 (0.4, 3.3)	151 (26, 242)

Extreme heat and cold represent temperature ranges at 97.5th percentile of temperature or above (≥ 29 °C), and at the 2.5th percentile of temperature or below (≤ − 6.5 °C), respectively. Moderate heat and cold represent temperature ranges between the MAT (22 °C) and the 97.5th or 2.5th percentile of temperature, respectively

*MAT* minimum admission temperature, *AF* attributable fraction, *AN* attributable number

The attributable risks varied among different subgroups. The overall estimated temperature-related burdens of asthma hospitalization were much higher in females (34.5%, 95% eCI 21.6, 44.2) than males (19.9%, 95% eCI 1.0, 33.5). The results were constant when further comparing the burdens resulting from different components of temperature between the two gender groups. Compared with the population aged ≥ 65 years old (26.0%, 95% eCI 7.7, 39.4), the total AF of non-optimum temperature was slightly higher in the population aged 19–64 years old (30.9%, 95% eCI 18.1, 40.7). The comparison between the two age groups was with little differences for the burdens attributable to moderate and extreme temperatures.

### Sensitivity analyses

The risk estimates did not substantially differ after changing the dfs for relative humidity (3–5), wind speed (3–5), and AQI (3–5), altering the df (3–5) of the natural cubic spline in the log scale for the lag-response space, and changing the maximum lag days (14 or 21) in the model (see Additional file 1: Tables S1–S3 and Additional file 2: Fig. S1). Besides, our results were still robust by applying different temperature metrics, including daily minimum temperature, daily maximum temperature and daily mean apparent temperature (see Additional file 1: Table S4 and Additional file 2: Figs. S2–S4).

## Discussion

Our results revealed that both low and high temperatures were significantly associated with increased risks of adult asthma hospitalizations. The associations were non-linear and followed U-shape curves in all the subgroups and the total population. Overall, a high fraction (29.1%) of adult asthma hospitalizations were attributable to non-optimum temperature over lag 0–30 days. Most of the hospitalization burden was attributable to moderate cold exposures. Females and younger patients were more susceptible to the short-term effects of extreme temperatures with greater burdens attributable to non-optimum temperatures. This is the first study estimating the attributable burden of adult asthma hospitalizations from ambient temperatures to the best of our knowledge.

We found that higher cumulative risks of adult asthma hospitalizations were related to both heat and cold exposures in Beijing, China. The extreme cold effect was higher than the extreme heat effect. Most previous studies only showed significant cold effects while no apparent association between heat exposures and asthma was detected. A similar study conducted in Shanghai, China reported that lower temperature (the 1st percentile of temperature relative to the median temperature) was associated with increased asthma hospitalizations with CRR = 1.79 (95% CI 1.18, 2.72) at lag 0–30 days [11]. Another study in Dongguan, China found that the CRR associated with extreme cold (the 5th percentile of temperature relative to the minimum morbidity temperature) for asthma outpatient visits was 1.04 (95% CI 1.00, 1.08) at lag 0–7 days [21]. On the other hand, a few studies have suggested positive associations between high temperatures and asthma hospitalizations. Lam et al. conducted a time-series study in Hong Kong and found that in hot seasons, the CRR associated with heat exposures (30 °C vs. 27 °C) was 1.19 (95% CI 1.06, 1.34) at lag 0–3 days [8]. The inconsistent results among various studies may be explained by differences in climate conditions, study designs and analytical approaches.

The intrinsic biological mechanisms are still unclear. The increased cold-related asthma hospitalizations may be associated with increasing bronchoconstriction [36], airway inflammation [37], mucus secretion [38] and decreasing effectiveness of immune responses [39] triggered by low temperatures directly. Moreover, cold temperatures can favor the transmission and survival of influenza viruses [40, 41], which may increase the risk of infection-related exacerbations of asthma. The possible mechanisms between heat exposures and asthma exacerbations point to bronchoconstriction mediated by the cholinergic reflex pathway [42] and airway inflammation aggravation through transient receptor potential channels [43]. Additionally, high temperatures play a key role in plants producing allergenic pollens with stronger allergenicity [4]. High concentrations of allergens, such as pollens and fungi in the air have been linked to the increased asthma severity in both children and adults [44]. On the other hand, people tend to spend more time indoors during extreme temperatures for obtaining better comfort, either using artificial heating during low ambient temperatures or using artificial cooling during high ambient temperatures. This extended stay indoors may increase the exposure to indoor molds, allergens or pollutants, which are known causes of asthma exacerbations [45–47].

Findings from our study showed that non-optimum temperatures were responsible for a substantial portion (29.1%) of adult asthma hospitalizations over lag 0–30 days. Most hospitalizations (20.3%) were attributed to the days with moderate cold temperatures. These results were consistent with several previous studies focusing on mortality [31, 35, 48]. However, Zhao et al. found that moderate heat exposure accounted for most of the morbidity burden of asthma in Dongguan, China [21]. The attributable burden for the temperature-asthma association may vary by distributions of days in different temperature ranges. Although extreme temperatures bring higher risk than moderate temperatures, the moderate cold days were the most in number in our study. Consequently, more attention should be paid to moderate cold when planning adaptation strategies and measures to reduce asthma hospitalizations.

Our subgroup analysis by gender showed that female patients with asthma were more vulnerable to ambient temperatures than male patients, which is similar to some epidemiological studies [12, 22]. The reasons for the discrepancy in the susceptibility of temperature-related asthma exacerbations by gender may point to bronchial hyperresponsiveness and estrogen in females [49]. As for subgroup analysis by age, we found that the younger population (19–64 years old) had higher risks of asthma hospitalizations attributable to ambient temperatures than the elderly, which was also shown in other prior studies [8, 14, 16]. Younger people were more vulnerable to temperature, possibly because they stay longer for work outdoors and are more likely to be exposed to abnormal temperatures.

There are some limitations to this study. Firstly, the temperature data were from fixed meteorological monitoring stations rather than individual exposure measurements, which may not reflect real exposures. Secondly, the data of some potential confounding factors, such as pollens, precipitation and thunderstorms, were unavailable for analysis. These factors are likely to impact the risk of asthma attacks and the number of hospitalizations [7]. Thirdly, since our study focused on the adult population in a single city, the extrapolation of our findings to other regions and the children population should be undertaken cautiously. Fourthly, mild asthma exacerbations treated in the outpatient setting or emergency department were not included in the study. We only focused on the association between ambient temperature and more severe asthma exacerbations requiring admission to a hospital ward, which may underestimate the effect of abnormal temperatures. Lastly, as an ecological study, the unit of analysis is a group of people instead of individuals [50]. Hence, the results should be interpreted with caution when applying to individuals. More comprehensive, individual-based epidemiological studies are needed in the future.

## Conclusions

This study provides evidence of the non-linear associations between ambient temperature and adult asthma hospitalizations, and the corresponding disease burden that is mainly attributable to moderate cold in Beijing, China. The vulnerable populations including the youngers and females, need to strengthen their awareness of the adverse impacts of both extreme heat and cold exposures. Our findings may have significant implications that exposures to high and low temperatures should be considered as potentially preventable triggers of asthma hospitalizations. In the context of climate change, such evidence is crucial for planning proper health risk education to the public, tailoring effective intervention strategies and evaluating the overall burden of asthma hospitalizations associated with abnormal temperatures.

## Supplementary Information

**Additional file 1: Table S1.** Lag-cumulative relative risks for total adult asthma hospitalizations associated with extreme heat exposure [97.5th percentile (29 °C) relative to MAT (22 °C)] and cold exposure [2.5th percentile (− 6 °C) relative to MAT] over lag 0–30 days using different degrees of freedom of confounders. **Table S2.** Lag-cumulative relative risks for adult asthma hospitalizations associated with extreme heat exposure [97.5th percentile (29 °C) relative to MAT (22 °C)] and cold exposure [2.5th percentile (− 6.5 °C) relative to MAT] over lag 0–30 days using different degrees of freedom of natural cubic spline in the log scale for the lag-response space. **Table S3.** Lag-cumulative relative risks for adult asthma hospitalizations associated with extreme cold exposure (2.5th percentile relative to MAT) and heat exposure (97.5th percentile relative to MAT) using different maximum lag days in the distributed lag non-linear model. **Table S4.** Lag-cumulative relative risks for adult asthma hospitalizations associated with extreme cold exposure (2.5th percentile relative to MAT) and heat exposure (97.5th percentile relative to MAT) over lag 0–30 days employing different temperature metrics.

**Additional file 2: Figure S1.** Lag-cumulative exposure–response associations between daily mean temperature and total adult asthma hospitalizations in Beijing, 2012–2015 using different maximum lag days in the distributed lag non-linear model. **Figure S2.** Lag-cumulative exposure–response associations between daily minimum temperature and total adult asthma hospitalizations in Beijing, 2012–2015. **Figure S3.** Lag-cumulative exposure–response associations between daily maximum temperature and total adult asthma hospitalizations in Beijing, 2012–2015. **Figure S4.** Lag-cumulative exposure–response associations between daily mean apparent temperature and total adult asthma hospitalizations in Beijing, 2012–2015.

## Data Availability

The datasets used and/or analyzed during the current study are available from the corresponding author on reasonable request.

## Abbreviations

AF: Attributable number
AN: Attributable fraction
AQI: Air quality index
CI: Confidence interval
CRR: Cumulative relative risk
df: Degrees of freedom
DLNM: Distributed lag non-linear model
eCI: Empirical confidence interval
ICD-10: The 10th version of the International Classification of Diseases
MAT: Minimum admission temperature
RH: Relative humidity
RR: Relative risk
WS: Wind speed
