# Supplementary material for: Associations between ambient temperature and adult asthma hospitalizations in Beijing, China: a time-stratified case-crossover study
Source: Respir Res. 2022 Feb 22;23:38. doi: 10.1186/s12931-022-01960-8 (PMC8862352; doi:10.1186/s12931-022-01960-8)
Supplement: Supplementary file 1 — Additional file 1: Table S1. Lag-cumulative relative risks for total adult asthma hospitalizations associated with extreme heat exposure [97.5th percentile (29 °C) relative to MAT (22 °C)] and cold exposure [2.5th percentile (− 6 °C) relative to MAT] over lag 0–30 days using different degrees of freedom of confounders. Table S2. Lag-cumulative relative risks for adult asthma hospitalizations associated with extreme heat exposure [97.5th percentile (29 °C) relative to MAT (22 °C)] and cold exposure [2.5th percentile (− 6.5 °C) relative to MAT] over lag 0–30 days using different degrees of freedom of natural cubic spline in the log scale for the lag-response space. Table S3. Lag-cumulative relative risks for adult asthma hospitalizations associated with extreme cold exposure (2.5th percentile relative to MAT) and heat exposure (97.5th percentile relative to MAT) using different maximum lag days in the distributed lag non-linear model. Table S4. Lag-cumulative relative risks for adult asthma hospitalizations associated with extreme cold exposure (2.5th percentile relative to MAT) and heat exposure (97.5th percentile relative to MAT) over lag 0–30 days employing different temperature metrics. [file 12931_2022_1960_MOESM1_ESM.docx]

**Additional Tables**

| Confounders | df | Cold effect | Heat effect |
| --- | --- | --- | --- |
|  |  | CRR (95% CI) | CRR (95% CI) |
| Relative humidity | 3 | 2.35 (1.60, 3.47)^*^ | 2.08(1.55, 2.80)^*^ |
|  | 4^#^ | 2.32 (1.57, 3.42)^*^ | 2.04 (1.52, 2.74)^*^ |
|  | 5 | 2.36 (1.60, 3.47)^*^ | 2.05 (1.53, 2.76)^*^ |
| Wind speed | 3^#^ | 2.32 (1.57, 3.42)^*^ | 2.04 (1.52, 2.74)^*^ |
|  | 4 | 2.32 (1.57, 3.41)^*^ | 2.04 (1.52, 2.74)^*^ |
|  | 5 | 2.29 (1.56, 3.37)^*^ | 2.02 (1.51, 2.72)^*^ |
| AQI | 3 | 2.29 (1.55, 3.37)^*^ | 2.08 (1.55, 2.79)^*^ |
|  | 4^#^ | 2.32 (1.57, 3.42)^*^ | 2.04 (1.52, 2.74)^*^ |
|  | 5 | 2.31 (1.57, 3.41)^*^ | 2.03 (1.51, 2.73)^*^ |

**Table S1** Lag-cumulative relative risks for total adult asthma hospitalizations associated with extreme heat exposure [97.5^th^ percentile (29 ℃) relative to MAT (22 ℃)] and cold exposure [2.5^th^ percentile (-6 ℃) relative to MAT] over lag 0-30 days using different degrees of freedom of confounders

MAT, minimum admission temperature; AQI, Air quality index; df, degree of freedom; CRR, cumulative relative risk; CI, confidence interval

^#^Used in the study

^*^*p*<0.05

**Table S2** Lag-cumulative relative risks for adult asthma hospitalizations associated with extreme heat exposure [97.5^th^ percentile (29 °C) relative to MAT (22°C)) and cold exposure [2.5^th^ percentile (-6.5 °C) relative to MAT] over lag 0-30 days using different degrees of freedom of natural cubic spline in the log scale for the lag-response space

| df | Group | Cold effect | Heat effect |
| --- | --- | --- | --- |
|  |  | CRR (95%CI) | CRR (95%CI) |
| 3^#^ | Total | 2.32 (1.57, 3.42)^*^ | 2.04 (1.52, 2.74)^*^ |
|  | Male | 1.64 (0.94, 2.84) | 1.47 (0.99, 2.21) |
|  | Female | 2.90 (1.85, 4.54)^*^ | 2.60 (1.82, 3.70)^*^ |
|  | 19-64 years old | 2.46 (1.56, 3.89)^*^ | 2.39 (1.71, 3.34)^*^ |
|  | ≥65 years old | 2.11 (1.22, 3.64)^*^ | 1.56 (1.00, 2.43)^*^ |
| 4 | Total | 2.30 (1.56, 3.40)^*^ | 2.03 (1.51, 2.73)^*^ |
|  | Male | 1.64 (0.95, 2.86) | 1.47 (0.98, 2.20) |
|  | Female | 2.85 (1.81, 4.47)^*^ | 2.58 (1.81, 3.67)^*^ |
|  | 19-64 years old | 2.44 (1.54, 3.85)^*^ | 2.37 (1.69, 3.32)^*^ |
|  | ≥65 years old | 2.10 (1.21, 3.64)^*^ | 1.56 (1.00, 2.42)^*^ |
| 5 | Total | 2.26 (1.53, 3.35)^*^ | 2.03 (1.50, 2.74)^*^ |
|  | Male | 1.63 (0.93, 2.83) | 1.41 (0.93, 2.12) |
|  | Female | 2.78 (1.77, 4.38)^*^ | 2.65 (1.85, 3.79)^*^ |
|  | 19-64 years old | 2.37 (1.49, 3.75)^*^ | 2.32 (1.65, 3.27)^*^ |
|  | ≥65 years old | 2.11 (1.22, 3.67)^*^ | 1.61 (1.03, 2.52)^*^ |

MAT, minimum admission temperature; df, degree of freedom; CRR, cumulative relative risk; CI, confidence interval

^#^Used in the study

^*^*p*<0.05

**Table S3** Lag-cumulative relative risks for adult asthma hospitalizations associated with extreme cold exposure (2.5^th^ percentile relative to MAT) and heat exposure (97.5^th^ percentile relative to MAT) using different maximum lag days in the distributed lag non-linear model

MAT, minimum admission temperature; df, degree of freedom; CRR, cumulative relative risk; CI, confidence interval

^#^Used in the study

^*^*p*<0.05

| Maximum lag days | Group | Cold effect | Heat effect |
| --- | --- | --- | --- |
|  |  | CRR (95%CI) | CRR (95%CI) |
| 14 | Total | 1.93 (1.37, 2.72)^*^ | 1.67 (1.29, 2.18)^*^ |
|  | Male | 1.48 (0.92, 2.39) | 1.31 (0.92, 1.87) |
|  | Female | 2.31 (1.55, 3.44)^*^ | 2.02 (1.47, 2.77)^*^ |
|  | 19-64 years old | 2.13 (1.42, 3.18)^*^ | 1.83 (1.35, 2.46)^*^ |
|  | ≥65 years old | 1.68 (1.04, 2.72)^*^ | 1.45 (0.98, 2.15) |
| 21 | Total | 2.19 (1.51, 3.18)^*^ | 1.89 (1.43, 2.49)^*^ |
|  | Male | 1.62 (0.96, 2.73) | 1.37 (0.94, 1.99) |
|  | Female | 2.67 (1.73, 4.13)^*^ | 2.41 (1.73, 3.35)^*^ |
|  | 19-64 years old | 2.44 (1.57, 3.78)^*^ | 2.10 (1.53, 2.88)^*^ |
|  | ≥65 years old | 1.87 (1.11, 3.17)^*^ | 1.57 (1.04, 2.37)^*^ |
| 30^#^ | Total | 2.32 (1.57, 3.42)^*^ | 2.04 (1.52, 2.74)^*^ |
|  | Male | 1.64 (0.94, 2.84) | 1.47 (0.99, 2.21) |
|  | Female | 2.90 (1.85, 4.54)^*^ | 2.60 (1.82, 3.70)^*^ |
|  | 19-64 years old | 2.46 (1.56, 3.89)^*^ | 2.39 (1.71, 3.34)^*^ |
|  | ≥65 years old | 2.11 (1.22, 3.64)^*^ | 1.56 (1.00, 2.43)^*^ |

| Metrics  (MAT) | Group | Cold effect | Heat effect |
| --- | --- | --- | --- |
|  |  | CRR (95%CI) | CRR (95%CI) |
| Tmean^#^  (22 ℃) | Total | 2.32 (1.57, 3.42)^*^ | 2.04 (1.52, 2.74)^*^ |
|  | Male | 1.64 (0.94, 2.84) | 1.47 (0.99, 2.21) |
|  | Female | 2.90 (1.85, 4.54)^*^ | 2.60 (1.82, 3.70)^*^ |
|  | 19-64 years old | 2.46 (1.56, 3.89)^*^ | 2.39 (1.71, 3.34)^*^ |
|  | ≥65 years old | 2.11 (1.22, 3.64)^*^ | 1.56 (1.00, 2.43)^*^ |
| Tmin  (17 ℃) | Total | 2.21 (1.44, 3.41)^*^ | 2.02 (1.52, 2.69)^*^ |
|  | Male | 1.52 (0.82, 2.80) | 1.45 (0.98, 2.13) |
|  | Female | 2.82 (1.71, 4.67)^*^ | 2.61 (1.84, 3.69)^*^ |
|  | 19-64 years old | 2.27 (1.36, 3.80)^*^ | 2.27 (1.63, 3.14)^*^ |
|  | ≥65 years old | 2.12 (1.16, 3.89)^*^ | 1.66 (1.08, 2.55)^*^ |
| Tmax  (28 ℃) | Total | 2.45 (1.69, 3.56)^*^ | 2.13 (1.50, 3.02)^*^ |
|  | Male | 1.68 (0.99, 2.85) | 1.50 (0.92, 2.43) |
|  | Female | 3.14 (2.04, 4.83)^*^ | 2.74 (1.81, 4.14)^*^ |
|  | 19-64 years old | 2.58 (1.67, 3.99)^*^ | 2.60 (1.75, 3.88)^*^ |
|  | ≥65 years old | 2.23 (1.31, 3.79)^*^ | 1.52 (0.91, 2.56) |
| ATmean  (17.9 ℃) | Total | 3.04 (1.95, 4.74)^*^ | 2.43 (1.79, 3.29)^*^ |
|  | Male | 1.95 (1.03, 3.67)^*^ | 1.72 (1.13, 2.60) |
|  | Female | 4.03 (2.40, 6.76)^*^ | 3.13 (2.17, 4.51)^*^ |
|  | 19-64 years old | 3.25 (1.92, 5.50)^*^ | 2.91 (2.05, 4.12)^*^ |
|  | ≥65 years old | 2.70 (1.44, 5.05)^*^ | 1.80 (1.14, 2.83)^*^ |

**Table S4** Lag-cumulative relative risks for adult asthma hospitalizations associated with extreme cold exposure (2.5^th^ percentile relative to MAT) and heat exposure (97.5^th^ percentile relative to MAT) over lag 0-30 days employing different temperature metrics

MAT, minimum admission temperature; Tmean, daily mean temperature; Tmin, daily minimum temperature; Tmax, daily maximum temperature; ATmean, daily mean apparent temperature; CRR, cumulative relative risk; CI, confidence interval;

^#^Used in the study;

^*^*p*<0.05
