# Supplementary material for: Associations between ambient temperature and adult asthma hospitalizations in Beijing, China: a time-stratified case-crossover study
Source: Respir Res. 2022 Feb 22;23:38. doi: 10.1186/s12931-022-01960-8 (PMC8862352; doi:10.1186/s12931-022-01960-8)
Supplement: Supplementary file 2 — Additional file 2: Figure S1. Lag-cumulative exposure–response associations between daily mean temperature and total adult asthma hospitalizations in Beijing, 2012–2015 using different maximum lag days in the distributed lag non-linear model. Figure S2. Lag-cumulative exposure–response associations between daily minimum temperature and total adult asthma hospitalizations in Beijing, 2012–2015. Figure S3. Lag-cumulative exposure–response associations between daily maximum temperature and total adult asthma hospitalizations in Beijing, 2012–2015. Figure S4. Lag-cumulative exposure–response associations between daily mean apparent temperature and total adult asthma hospitalizations in Beijing, 2012–2015. [file 12931_2022_1960_MOESM2_ESM.docx]

**Additional Figures**


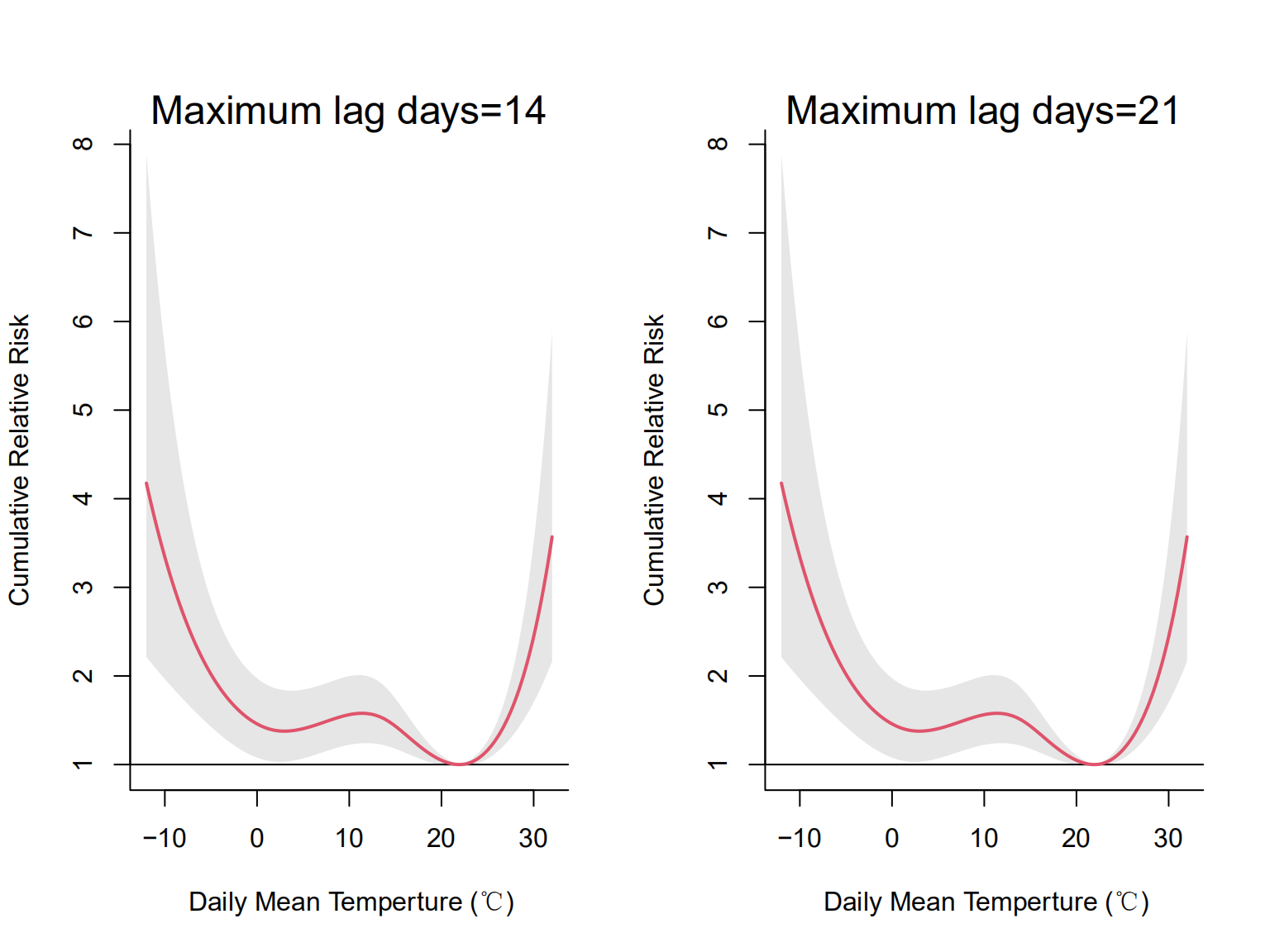


**Figure S1** Lag-cumulative exposure-response associations between daily mean temperature and total adult asthma hospitalizations in Beijing, 2012-2015 using different maximum lag days in the distributed lag non-linear model


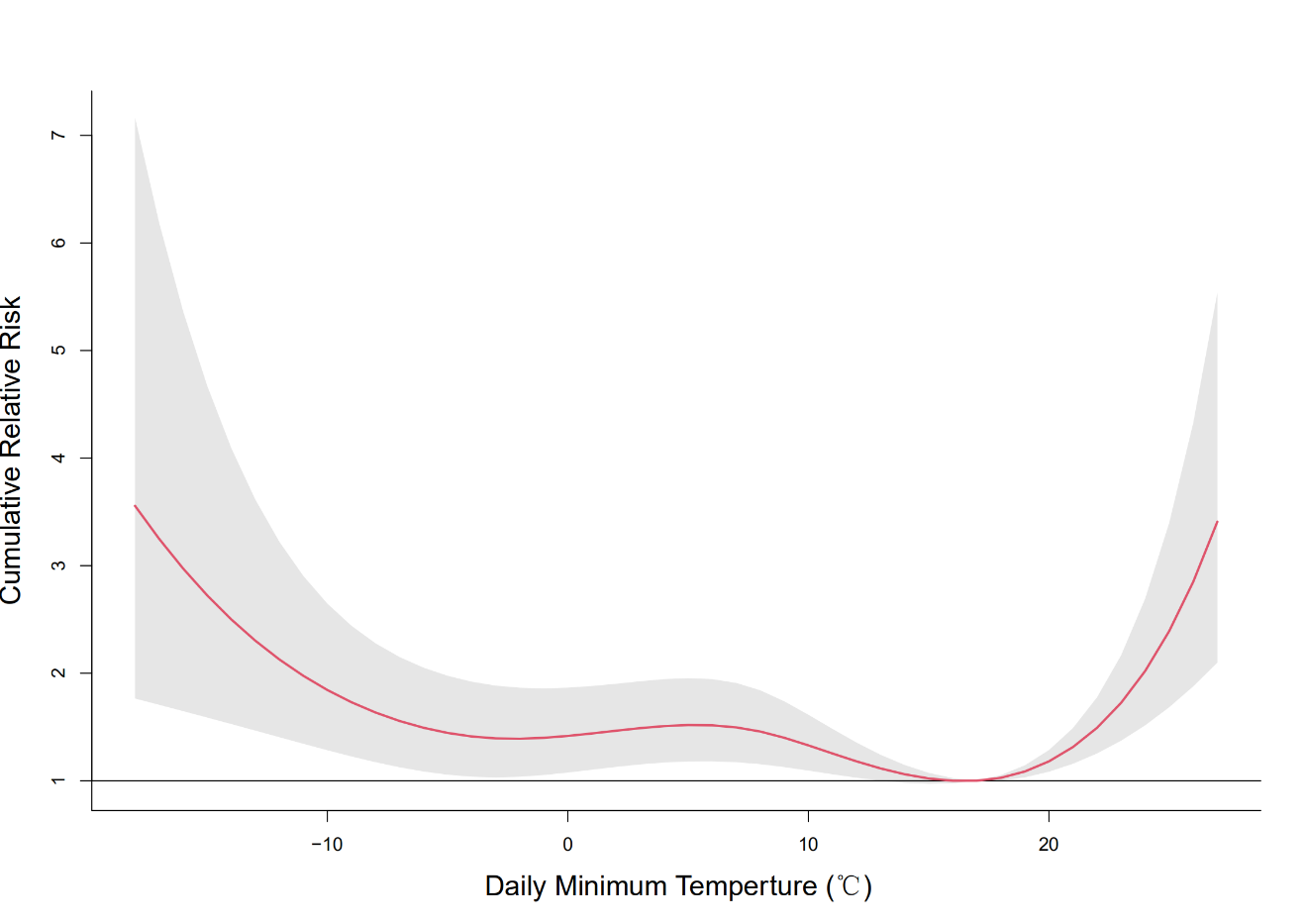
**Figure S2** Lag-cumulative exposure-response associations between daily minimum temperature and total adult asthma hospitalizations in Beijing, 2012-2015


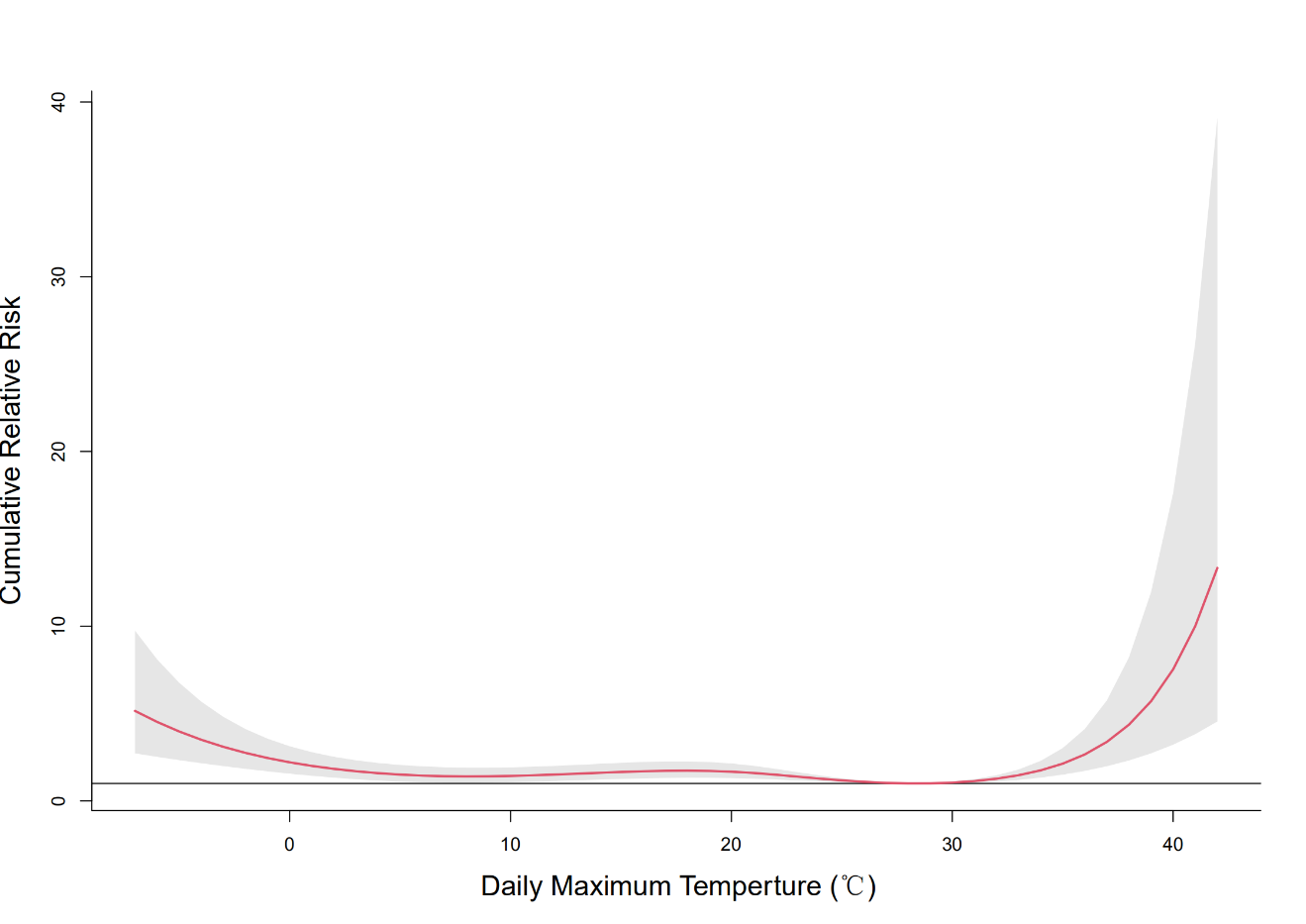
**Figure S3** Lag-cumulative exposure-response associations between daily maximum temperature and total adult asthma hospitalizations in Beijing, 2012-2015


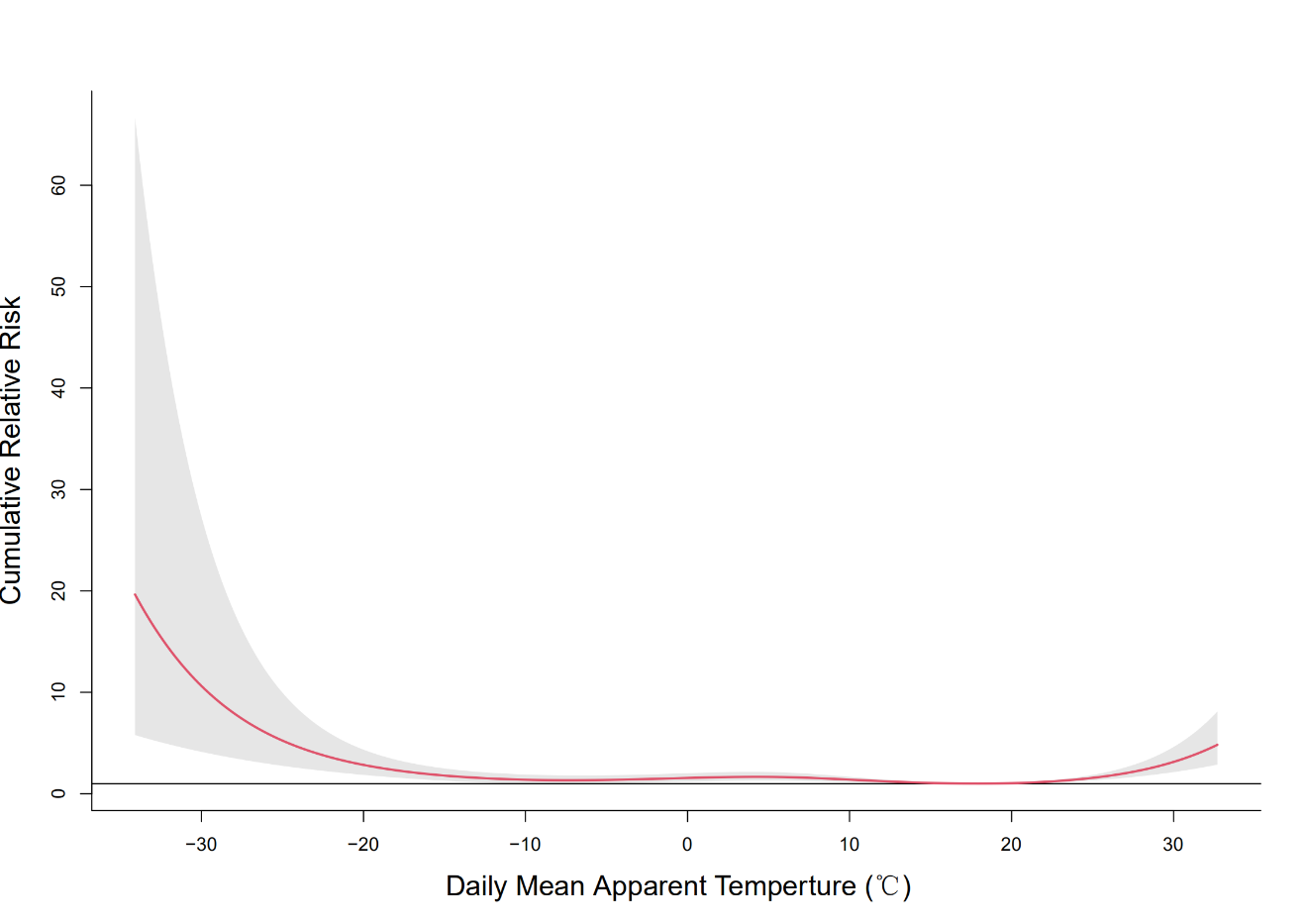
**Figure S4** Lag-cumulative exposure-response associations between daily mean apparent temperature and total adult asthma hospitalizations in Beijing, 2012-2015
